# miR-155 as a Biomarker in B-Cell Malignancies

**DOI:** 10.1155/2016/9513037

**Published:** 2016-05-16

**Authors:** Hanne Due, Pernille Svendsen, Julie Støve Bødker, Alexander Schmitz, Martin Bøgsted, Hans Erik Johnsen, Tarec Christoffer El-Galaly, Anne Stidsholt Roug, Karen Dybkær

**Affiliations:** ^1^Department of Haematology, Aalborg University Hospital, Hobrovej 18-22, 9100 Aalborg, Denmark; ^2^Department of Haematology, Aarhus University Hospital, Tage-Hansens Gade 2, 8000 Aarhus C, Denmark; ^3^Department of Mathematical Sciences, Aalborg University, Fredrik Bajers Vej 5, 9100 Aalborg, Denmark; ^4^Department of Clinical Medicine, Aalborg University, Fredrik Bajers Vej 5, 9100 Aalborg, Denmark; ^5^Clinical Cancer Research Center, Aalborg University Hospital, Hobrovej 18-22, 9100 Aalborg, Denmark

## Abstract

MicroRNAs have the potential to be useful biomarkers in the development of individualized treatment since they are easy to detect, are relatively stable during sample handling, and are important determinants of cellular processes controlling pathogenesis, progression, and response to treatment of several types of cancers including B-cell malignancies. miR-155 is an oncomiR with a crucial role in tumor initiation and development of several B-cell malignancies. The present review elucidates the potential of miR-155 as a diagnostic, prognostic, or predictive biomarker in B-cell malignancies using a systematic search strategy to identify relevant literature. miR-155 was upregulated in several malignancies compared to nonmalignant controls and overexpression of miR-155 was further associated with poor prognosis. Elevated expression of miR-155 shows potential as a diagnostic and prognostic biomarker in diffuse large B-cell lymphoma and chronic lymphocytic leukemia. Additionally,* in vitro* and* in vivo* studies suggest miR-155 as an efficient therapeutic target, supporting its oncogenic function. The use of inhibiting anti-miR structures indicates promising potential as novel anticancer therapeutics. Reports from 53 studies prove that miR-155 has the potential to be a molecular tool in personalized medicine.

## 1. Introduction

Personalized medicine is a new principle that aims at tailoring medical treatment of the individual patients and thereby ending the current “one-fits-all” strategy. Today's cancer diagnostics are typically based on clinical findings, morphology, histology, cytogenetic, immune-phenotyping, and molecular genetic data, but still identification of the molecular pathways driving tumorigenesis often fails [1]. Different B-cell malignancies share common molecular pathways, which is why they may benefit from the same pathway-specific targeted treatment. Additionally, tumor subtypes within one disease entity can be characterized by distinct molecular pathogenesis markers as genetic aberrations or transcription phenotypic markers but still be treated alike causing inefficient expenditure treatment regardless of potential subgroup-specific treatment efficiency. The aim of personalized medicine is to drive the development of a more accurate classification of disease, defined by molecular pathogenesis ultimately enhancing diagnosis and treatment by the use of easy detectable biomarkers [2].

Biomarkers are defined as objective indicators of biological processes, pathogenic processes, or pharmacological response to a therapeutic intervention [3]. Diagnostic biomarkers identify the presence of disease and differentiate normal from malignant or distinguish different diagnoses or progression stages. Prognostic biomarkers provide information about clinical outcome for a class of patients when given a specific treatment, whereas predictive biomarkers provide information on how patients are expected to respond to a drug or treatment regimen. Most importantly, all biomarkers should add further information to present clinical tools. In order to ensure accurate stratification, ideal biomarkers need to be easy to detect and provide both high sensitivity and specificity [4].

MicroRNAs (miRNAs) have been demonstrated to possess biomarker potential in multiple diseases [5], both individually and when combined in signature profiles [6–9]. miRNAs are short noncoding RNAs of 20–22 nucleotides that function to regulate gene expression at the posttranscriptional level. They play fundamental roles in the regulation of cellular proliferation, differentiation, and apoptosis [10]. miRNAs are deregulated in many types of cancer, including B-cell malignancies, where they can function as oncogenes, favoring initiation and progression of cancers, or as tumor suppressors, preventing tumorigenesis [11, 12]. One of the most widely studied miRNAs in B-cell malignancies is the oncogenic miR-155, transcribed from a noncoding RNA BIC (B-cell Integration Cluster). miR-155 biogenesis is only briefly summarized since it has recently been extensive and thoroughly reviewed by others [13, 14]. At normal physiologic conditions, miR-155 is a crucial player in hematopoiesis, the immune response, and inflammation [15–18]. It has been found to be upregulated in several types of cancers [19] and has shown specific importance in the pathogenesis of B-cell malignancies. The oncogenic function of miR-155 can be explained by its target genes and the involved underlying molecular pathways presented in Table 1. Overexpression of miR-155 in mice results in development of lymphoproliferative diseases, while subsequent withdrawal leads to remission [20]. Thus, miR-155 is suggested to be a future treatment target. A high number of studies have investigated its potential as a biomarker in several B-cell malignancies, though conflicting results have been presented. To elaborate and assess the potential of miR-155 as a diagnostic, prognostic, and predictive biomarker or target of novel treatments in B-cell malignancies as a part of personalized medicine, we systematically reviewed the existing literature.

## 2. Materials and Methods

This review was prepared according to the Preferred Reporting for Systematic Reviews and Meta-Analyses (PRISMA) Guidelines [21].

### 2.1. Literature Search

PubMed and EMBASE were systematically searched for eligible articles. The search terms used in both databases are provided in Table S1 in Supplementary Material available online at http://dx.doi.org/10.1155/2016/9513037. The search was finalized on November 18, 2015. Additional studies were identified by scanning reference lists of articles. The screening process was performed by two reviewers by reading titles and abstracts, while the eligibility of full-texts was assessed in the same manner.

### 2.2. Inclusion and Exclusion Criteria

Studies were included in the analysis if fulfilling the following inclusion criteria: (1) concerning miR-155 expression as a biomarker or target of chemotherapeutic treatment, (2) focusing on B-cell malignancies, (3) analyzing patient samples, (4) original research articles or letters, and (5) results published in English. Articles were excluded if the present disease was reported in ≤2 independent studies.

### 2.3. Analysis

Data concerning the specific disease, cohort size, sample type, study design of miRNA selection, analytical method, and outcome was extracted manually, and studies were grouped according to the investigated biomarker properties of miR-155 (i.e., diagnostic, prognostic, and/or predictive). Studies exploiting miR-155 as a therapeutic target were described according to their methods (e.g.,* in vitro*/*in vivo*), outcomes, and impact of their findings.

## 3. Results

The systematic search revealed 606 articles, which were screened by reading title and abstract. A total of 126 full-texts were assessed, and 53 of these studies were included in the review, presented in Figure S1. All included articles were published in peer-reviewed scientific journals. Forty-eight of the included articles investigated the expression of miR-155 as either a diagnostic, prognostic, or predictive tool in the management of several diseases, though we excluded studies of specific diseases represented by two or less papers. Five papers exploited miR-155 as a potential target in the treatment of B-cell malignancies. As presented in Table 2, the diagnostic potential of deregulated miR-155 expression has been widely investigated in diffuse large B-cell lymphoma (DLBCL), Burkitt's lymphoma (BL), Hodgkin's lymphoma (HL), mucosa-associated lymphoid tissue lymphoma (MALT), follicular lymphoma (FL), splenic marginal zone lymphoma (SMZL), and chronic lymphatic leukemia (CLL). Additionally, the prognostic potential of miR-155 has been investigated in several studies of DLBCL and CLL, whereas the predictive potential has not been thoroughly studied. Hence, the following report will primarily focus on DLBCL and CLL, as the biomarker potential of miR-155 has been reported more extensively in these malignancies. Malignancies represented by few conflicting studies are not discussed further in the review.

### 3.1. Diffuse Large B-Cell Lymphoma

DLBCL is a highly aggressive disease representing a clinically, morphologically, and genetically heterogeneous group of non-Hodgkin lymphomas. Despite the treatment improvements by inclusion of rituximab, up to 40% of the patients eventually die from relapsing or refractory disease [30, 31]. In general, detection of precancerous lesions and early stage cancers is crucial to reducing the disease mortality. Early detection of DLBCL may likewise permit treatment of early stages, which can prevent disease-related deaths. Thus, it is necessary to identify new diagnostic biomarkers for clinical use. Through the systematic search, we found 18 studies focusing on the expression of miR-155 as a diagnostic marker in DLBCL, presented in Table 2. All studies comparing the expression level in DLBCL patients to healthy controls found a significant upregulation of miR-155 in DLBCL. The mean fold-change values span from 3 to 19 [32–36] and Fang et al. reported a cutoff value of 0.0022 and a sensitivity and specificity at 83% and 65%, respectively [37]. Distinction between non-Hodgkin lymphomas, such as DLBCL, FL, and BL, can be difficult due to great molecular and clinical heterogeneity. In addition to the need for early detection, new biomarkers should also improve the accuracy of lymphoma diagnosis and decisions of therapeutics. Three studies found miR-155 higher expressed in DLBCL compared to BL patients illustrating miR-155 as a potential diagnostic biomarker [38–40]. However, studies comparing DLBCL and FL showed no significant differential expression [33, 34, 36]. Blood samples were analyzed in two studies [35, 37] and frozen tumor tissue and FFPE tissue in the remaining.

Using gene-expression profiling, DLBCL can be divided into the two molecular subtypes: germinal center B-cell-like (GCB) and activated B-cell-like (ABC) [41, 42]. The subtypes present different clinical outcome with GCB patients having a 5-year survival rate of 60% compared to 35% for those patients with ABC DLBCL [43]. In order to simplify and make accessible in a routine clinical setting, the molecular subtype identification has been implemented in several centers by the use of immunohistochemical (IHC) analysis resulting in DLBCL subtyping into GCB/non-GCB or GCB/ABC [42, 44, 45]. In this systematic review, we identified 15 articles evaluating the prognostic impact of miR-155, of which 13 studied the correlation between miR-155 and the molecular subtypes (Table 3). miR-155 was upregulated in the ABC subtype in nine studies while the remaining three did not find significant differential expression between the subgroups. Since patients classified as ABC exhibit an adverse prognosis, miR-155 holds the potential as prognostic marker.

Conflicting results were found in studies investigating the association of miR-155 expression and clinical outcome. Zhong et al. stratified patients according to high or low expression of miR-155 with a cutoff value at 3.98 and sensitivity and specificity value at 80% and 58.5%, respectively [32]. High miR-155 expression was significantly associated with adverse prognosis, which was also reported by Iqbal et al. using similar expression level stratification [39]. Zhong et al. further demonstrated that miR-155 and the international prognostic index (IPI) were statistically significant independent prognostic factors [32]. Contradictorily, other studies found that miR-155 expression did not correlate with DLBCL outcome [36, 49, 75]. Surprisingly, Jung and Aguiar observed that high expression of miR-155 was associated with improved prognosis exclusively within the ABC subgroup [75]. Zhong et al. showed predictive potential of miR-155, where patients treated with CHOP (cyclophosphamide, doxorubicin, vincristine, and prednisone) were compared to a cohort of R-CHOP (addition of rituximab) treated patients [32]. Interestingly, high expression of miR-155 improved clinical outcome in patients treated with R-CHOP compared to CHOP. This difference was not seen in patients with low miR-155 expression, suggesting that miR-155 has the potential to guide treatment with rituximab [32]. Additionally, Iqbal et al. reported that high miR-155 expression significantly correlated with R-CHOP treatment failure, suggesting a potential role as predictive biomarker [39]. This finding was supported by* in vitro* studies showing that high miR-155 expression sensitizes cells to synthetic Akt inhibitors, suggesting a novel treatment option for resistant DLBCL patients [39].

### 3.2. Chronic Lymphocytic Leukemia

CLL is characterized by clonal proliferation of mature B-cells accumulating in the peripheral blood, bone marrow, lymph nodes, and spleen [76]. Despite its prevalence, no cure exists and patients are treated with various chemotherapeutic drugs at the presence of progressive or symptomatic disease [76]. Several studies investigated the expression level of miR-155 in samples from CLL patients as compared to healthy controls, Table 2. Interestingly, none of these studies aimed at establishing new diagnostic tools but focused on elaborating the molecular pathogenesis of the disease or use preliminary diagnostic signatures of deregulated miRNAs to single out potential prognostic biomarkers. In all studies and irrespective of the analytical technique, miR-155 was upregulated in CLL compared to healthy controls. A few studies reported a mean fold-change of miR-155 expression in the range of 2–5, while individual samples showed great varying fold-changes [67, 69–71].

The prognostic potential of miR-155 expression in CLL was studied more extensively and showed varying results, as presented in Table 3. Particularly, favorable factors showed conflicting associations with miR-155 expression. CLL is usually described by many different prognostic factors, such as clinical staging systems (Rai and Binet), somatic hypermutation of the immunoglobulin heavy chain variable region (IgHV), surface CD38 expression, expression of zeta-associated protein 70 (ZAP-70), or chromosomal abnormalities (17p, 13q, 11q, and trisomy 12) [76]. Relating miR-155 expression to the individual prognostic factors revealed no correlation. However, studies often focused on different prognostic factors, making concise comparisons and conclusions impossible. In patients with 17p deletion, the expression of miR-155 was either upregulated or nonsignificantly differentiated between groups [77, 78]. miR-155 was overexpressed in both studies investigating 11q deletions, though patients with trisomy 12 had either downregulated or unaffected expression [66, 77]. ZAP-70 expression was related to the upregulation of miR-155 in two studies, while there was no association in four other studies [72–74, 79–81]. miR-155 was either downregulated or unaffected in patients with IgHV mutations [68, 71–74, 79–81], while 13q deletions were associated with high, low, and unaffected expression levels [66, 71, 77, 82]. Thus, the studies generally showed no specific correlation between miR-155 expression and favorable (13q deletion and IgHV mutation) or unfavorable (17p, 11q deletion, trisomy 12 and ZAP-70 expression) prognostic factors.

When the elevated expression of miR-155 was directly correlated with survival data, high expression was not consistently associated with poor prognosis. However, studies used different outcome measures, complicating the assessment of the prognostic potential of miR-155. Furthermore, studies failed to report specific treatment regimens and treatment homogeneity of their cohorts. Ferrajoli et al. investigated the survival of CLL patients stratified according to high or low plasma miR-155 expression in two different cohorts [74]. One cohort received treatment with the FCR regimen (fludarabine, cyclophosphamide, and rituximab), while the other cohort received single agent treatment with lenalidomide. High miR-155 expression was in both cohorts associated with poor treatment outcome estimated by clinical response assessment (RA) [74]. According to Lawrie et al. patients relapsing after treatment with fludarabine and rituximab either with or without alemtuzumab showed poor progression-free survival (PFS) when stratified according to high miR-155 expression [66]. It was further shown that monitoring miR-155 expression after treatment with ibrutinib could be an indicator of treatment failure. The expression of miR-155 decreased upon treatment, and patients whose expression rose above baseline during follow-up were prone to experience disease relapse [66].

Predictive biomarker potential of miR-155 has not been directly investigated, yet detection of 17p deletions by miR-155 as a surrogate marker could guide treatment decisions. Today, patients with 17p deletions are treated more aggressively due to poor prognostic results of front-line treatment with FCR [83]. As mentioned above, high miR-155 expression was associated with treatment failure in FCR and lenalidomide treated patients, suggesting potential use of miR-155 as a predictive biomarker [74].

### 3.3. Mucosa-Associated Lymphoid Tissue (MALT)

Extranodal marginal zone lymphomas (MALT lymphomas) are rare, low-grade B-cell lymphomas of mucosa-associated lymphoid tissue. Expression of miR-155 in MALT was found elevated in three out of three eligible studies [59–61]. Thorns et al. reported a stepwise increase in miR-155 expression from benign to malignant lymphoepithelial lesions [59]. Gastric MALT can be associated with chronic inflammation triggered by infection with* Helicobacter pylori* (*H. pylori*). Antibiotic treatment leads to complete remission in 60–80% of the patients; however improved identification of nonresponsive patients is needed to guide treatment [84]. The study by Saito et al. observed that resistant patients had a higher miR-155 level than cases showing complete remission, suggesting the potential of miR-155 as a predictive indicator [61].

### 3.4. Splenic Marginal Zone Lymphoma (SMZL)

SMZL is a rare form of small B-cell malignancy infiltrating the spleen, bone marrow, and peripheral blood. Three studies reported increased expression of miR-155 in diseased samples compared to controls [63–65]. Peveling-Oberhag et al. found a fold-change of miR-155 of 2.8 [63]. Additionally, Arribas et al. showed significantly increased miR-155 expression in SMZL spleen samples compared to nonmalignant samples from reactive spleens [64]. In contrast, the expression of miR-155 was downregulated in SMZL samples compared to spleens infiltrated by FL, CLL, and mantle cell lymphoma, though this change was not significant [64].

### 3.5. miR-155 as a Therapeutic Target

**Figure 1:**
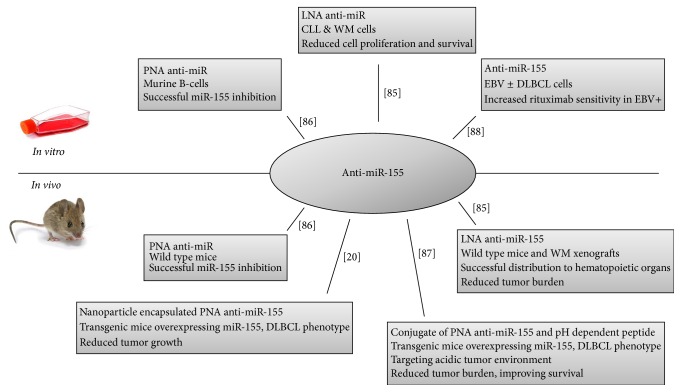
Studies (*n* = 5) exploiting miR-155 as a therapeutic target using anti-miR-155 structures. PNA, peptide nucleic acid; LNA, locked nucleic acid; CLL, chronic lymphocytic leukemia; WM, Waldenstrom macroglobulinemia; DLBCL, diffuse large B-cell lymphoma; EBV+, Epstein-Barr virus positive; EBV−, Epstein-Barr virus negative.

Due to the oncogenic function of miR-155 in especially B-cell malignancies, miR-155 holds potential as a target for future therapeutic interventions, exploited by five studies, Figure 1. Chemically modified synthetic oligonucleotides are efficient inhibitors of miRNAs* in vitro* and* in vivo*, improving systemic stability and binding affinity of the anti-miRNA [85, 86]. They bind the miRNA structure by complementary hybridization, preventing the miRNA from binding to its target mRNA. Usually, synthetic oligonucleotides such as PNA (peptide nucleic acid) and LNA (locked nucleic acid) are used. These RNA/DNA analogues are constructed by changing the nucleic acid backbone structures, and studies have proven their efficient inhibition of miR-155* in vitro* in murine B-cells and patient-derived CLL and Waldenstrom cell lines [85, 86]. Anti-miR-155 exposure resulted in decreased cell proliferation and survival of the CLL and Waldenstrom cells [85]. Evaluation of the systemic stability and efficacy was investigated in wild type mice and Waldenstrom xenografts [85, 86]. miR-155 expression was completely inhibited in the spleen upon injection of PNA anti-miR-155 in wild type mice [86]. Zhang et al. examined the distribution and intracellular uptake of fluorescence marked LNA anti-miR-155 in hematopoietic organs in wild type mice and Waldenstrom xenografts [85]. The anti-miR-155 was successfully delivered to cells in these specific organs. Additionally, anti-miR-155 intravenous administration resulted in decreased tumor burden in the Waldenstrom xenografts [85].

Drawbacks of systemic delivery are related to biological stability in the organism and intracellular uptake of the anti-miR. Babar et al. exploited the use of a nanoparticle-based delivery system of anti-miR-155 in a transgenic mouse model overexpressing miR-155 [20]. The nanoparticle encapsulated the anti-miR-155 structure to aid its stability and delivery. Additionally, coating of the nanoparticle with cell-penetrating peptides improved the intracellular uptake of anti-miR-155* in vivo* [20].

In order to use anti-miR-155 as therapeutics, challenges regarding nonspecific organ distribution have to be overcome. miR-155 is constitutively expressed in several tissues and has a crucial role in the function of the immune system [16, 17]. Thus, tumor specific distribution is warranted to avoid disruption of normal immunologic function, causing critical side effects. Cheng et al. showed a novel model for tumor specific distribution, utilizing tumor environment acidity, a hallmark of cancer [87]. They developed a conjugate of the anti-miR-155 structure and a pH-induced transmembrane structure peptide. The peptide has the ability to localize the acidic tumor microenvironment and at low pH, the peptide forms an inducible transmembrane helix promoting translocation of impermeable molecules across the cell membrane. Hereby the anti-miR-155 is efficiently delivered into the tumor cells causing reduced cell viability. Using the same miR-155 overexpressing transgene mice as Babar et al., the mice spontaneously developed lymphoma progressing from follicular hyperplasia to DLBCL. Administration of the anti-miR-155 conjugate at the time of tumor manifestation resulted in reduced tumor volume, suppressed metastatic spread of neoplastic cells, and improved survival compared to controls. High-dose administration to healthy mice showed absence of systemic toxicity, including maintenance of normal liver and kidney function [87]. Thus, this study introduces a novel model for using anti-miR as anticancer drug, having great impact on both targeted drug delivery and personalized medicine, since individual miR-155 expression levels are easily measured.

As mentioned, resistance to therapy is observed in 40% of patients with DLBCL and consequently, novel treatment options for resistant patients are needed [31]. Iqbal et al. reported treatment failure of R-CHOP in patients with high miR-155 expression and suggested Akt inhibitors as alternative therapeutics, since miR-155 activates this specific pathway [39]. The effect of Akt inhibitors was investigated in Epstein-Barr virus positive (EBV+) cell lines by Kim et al. [88]. Initially they found EBV+ cell lines to be resistant toward rituximab, having a phosphorylated Akt pathway, and simultaneous overexpression of miR-155. Akt inhibitors restored the sensitivity toward rituximab, and anti-miR-155 significantly reduced the cell survival upon rituximab exposure [88]. Thus, both Akt inhibitors and anti-miR-155 hold potential as add-on drugs to increase the response of DLBCL patients treated with R-CHOP.

### 3.6. Targets of miR-155

To understand how miR-155 act and identify the underlying molecular mechanisms driving tumorigenesis, many studies have investigated the target genes, of which some are listed in Table 1. Identification of targets and the involved pathways is important since it puts the biomarker into molecular perspective and additionally is crucial to understanding of the underlying molecular effects of using anti-miR-155 as an antineoplastic drug.

miR-155 was reported to target* SHIP1* and* C/EBPβ*, which are two important inhibitors of the IL-6 signaling pathway. Downregulation of these genes blocks B-cell differentiation and causes an improved cell survival due to activation of PI3K/Akt and MAPK pathway [22, 23]. Additionally, by targeting* HGAL*, a lymphocyte motility inhibitor, miR-155 promotes cell migration, which could contribute to a more aggressive disease [26]. Overexpression of miR-155 also leads to downregulation of* SMAD5*, a modulator of TGF-*β* signaling. miR-155 overexpression renders DLBCL cells resistant to growth inhibitory effects of TGF-*β* and BMP via defective p21 induction and impaired cell cycle arrest [27]. The two death domain containing genes* FADD* and* Ripk1* are also identified as target genes of miR-155. It is thus reasonable to assume that miR-155 targeting of these transcripts could lead to antiapoptotic effects [28].

## 4. Discussion

This review identified a total of 53 studies addressing the potential of miR-155 as putative biomarker or as a therapeutic target in B-cell malignancies. The results, presented in Tables 2, 3, and 4, display that miR-155 expression may function as a valuable tool in both diagnosis and prognostic evaluation of DLBCL patients and having prognostic impact in CLL as the results showed consistency across multiple studies. Few studies reported diagnostic potential of miR-155 expression in MALT, SMZL, FL, and HL. However, based on the limited number of studies and samples included in those, the significance needs reconfirmation in independent studies using larger cohorts.

The results of miR-155 as diagnostic marker of DLBCL were very consistent and independent of sample type, cohort size, and methodology. High expression of miR-155 enables stratification of DLBCL patients from healthy controls and BL patients, supporting its potential as a diagnostic tool. In contrast, lack of accuracy in differentiating DLBCL from FL patients was observed. This systematic review also presents evidence that miR-155 expression is associated with DLBCL molecular subtypes, even though some studies did not find a significant differential expression [47, 49, 50, 90]. Cohort sizes varied considerably across studies and generally the larger the cohort, the more valid the result. Studies reporting nonsignificant results stand out with small cohorts exemplified by Fischer et al. having 21 patients included compared to the cohort of 90 in the study by Zhong et al. [32, 47]. Another important matter that makes the studies less comparable is the fact that the subtype classification of the DLBCL patients into GCB and non-GCB/ABC is performed by IHC analysis using different staining strategies and interpretation algorithms. Additionally, IHC is difficult to standardize due to variation between laboratories, such as sample handling, antibodies utilized, and observers.

The most surprising observation was made by Jung and Aguiar, finding miR-155 overexpression association with improved outcome in ABC DLBCL [75]. The reason for this association is not immediately clear; however, they suggest that action of target genes contributes to the findings. Noteworthily, only 24 ABC patients are stratified into low or high miR-155 expression illustrating the need to expand and validate the data in order to trust the information [75].

miR-155 was significantly upregulated in all studies comparing CLL cases to healthy controls, indicating diagnostic potential. However, CLL is easily diagnosed in clinical cases from blood analysis, arguing against the need of a novel diagnostic biomarker for this disease [83]. miR-155 as prognostic marker in CLL is not unambitious. However, an association of miR-155 expression and favorable prognostic factors differed greatly between studies. This could be due to the individual different factors investigated such as specific deletions and mutations. Noteworthily, studies failed to report the specific treatment regimens giving potential bias because the prognosis is dependent on the effectiveness of the treatment. In addition, if patients did not receive the same treatment, the studies are less comparable. In general, high miR-155 expression was often associated with more aggressive disease and poor prognosis, though not significant across all studies. Ultimately, miR-155 expression was not consistently sufficient in stratifying CLL patients according to individual prognostic factors. In contrast, elevated miR-155 expression as an independent factor was associated with poor clinical outcome across studies, suggesting its potential as a direct prognostic biomarker in CLL.

Considering the fact that miR-155 is an oncomiR [11], the findings of high expression in the poor prognostic ABC subtype and the adverse prognostic impact of miR-155 expression on survival in DLBCL are consistent and in accordance with the observed association of high expression and more aggressive disease in CLL.

When analyzing the findings, it is also important to consider differences in methodology. Initial global microarray screenings were performed in several studies; however, they were not based on the same microarray models giving variations in the miRNA covering probes. The other widely used method is RT-qPCR which is based on another technique and relies on probes other than those used in microarray detection. The fold-change and accuracy of the studies therefore cannot be directly compared across studies but concordance of upregulated miR-155 and pure outcome independent of platform supports the robustness of the association. In several studies, a training cohort is utilized to identify miR-155 as potential diagnostic or prognostic tool and subsequently a validation cohort is analyzed to test and validate the result, increasing the significance of the findings. Others exploit the same cohort but validate the result using a different detection technique. Both approaches strengthen the observations and make the findings more valid.

Different sample types have potential to cause conflicting results. Studies regarding diagnostic evaluation of miR-155 in DLBCL analyze blood samples, formalin-fixed/paraffin-embedded tissues, and frozen tissues; however, no inconsistency is observed, indicating stable expression and robust detection of miR-155 despite sample types, preparation, and storage. Each sample type has different advantages. FFPE tissue samples are the most abundant available archival material and miRNA can successfully be isolated from processed formalin-fixed material, due to miRNAs relative resistance toward RNase degradation. Using RT-qPCR and microarray analysis, similar results of miRNA expression are found in FFPE and frozen material [93]. Lawrie et al. and Fang et al. studied miR-155 expression in blood samples to investigate the potential as noninvasive biomarker [35, 37]. Search for noninvasive biomarkers for diagnosis, prognosis, and monitoring of cancers has long been the goal of clinical research.

A guideline for Strengthening the Reporting of Observational studies in Epidemiology-Molecular Epidemiology (STROBE-ME) has been proposed, though several studies included in this review failed to report their investigations thoroughly (e.g., sample types, storage, and handling). Additionally, the studies included in this review differed in their aims, outcomes measures, and methods, complicating the general comparison of the studies and rendering a statistical meta-analysis impossible. The validity of this systematic review is improved by the fact that PRISMA guidelines are met and that the search strategy encompassed MESH/ENTRY terms and free text words.

Introducing new potential biomarkers into the clinic holds great difficulties and challenges. Therefore, the Early Detection Research Network (EDRN) has suggested a systematic approach guiding the process of biomarker development similar to the clinical stages of drug development [94]. Phase 1 includes preclinical investigations, where tumor tissue is compared to healthy controls in order to identify differential characteristics. A clinical biomarker assay is developed and tested in phase 2, including evaluation of the biomarkers ability to distinguish subjects with cancer from those without cancer. Phase 3 is a retrospective investigation of the biomarkers ability to detect presence of disease before it is clinically diagnosed, whereas phase 4 evaluates biomarker properties in a prospective follow-up study. Finally, phase 5 evaluates whether the biomarker and early diagnosis improved the overall benefit for the screened population. Although the guideline focuses on developing diagnostic biomarkers, the structure is potentially valuable for prognostic and predictive biomarkers as well. All included diagnostic studies of CLL were consistent with phase 1 investigations, though as mentioned before a new diagnostic biomarker of CLL would hold limited clinical use. In addition, prognostic investigations of miR-155 in CLL could be described as phase 1 investigations, where a biomarker assay and assessment are still missing. Studies reporting miR-155 as potential diagnostic biomarker of DLBCL are all on the early phases of diagnostic biomarker development as well, which is why clinical implementation will require further studies at higher developmental phases. Only Fang et al. reported evaluation of miR-155's ability to distinguish DLBCL patients from healthy controls [37]. Thus, miR-155 cannot be considered as a diagnostic biomarker in clinical use at short term.

In order to implement the concept of personalized medicine, new molecular biomarkers need to be established to improve early diagnosis, patient stratification according to high-risk patients, and predictions of treatment response. According to the present assessment, miR-155 could hold potential as a novel diagnostic biomarker in several B-cell malignancies, including DLBCL and CLL. However, miR-155 still needs to move through the remaining biomarker developmental steps and evaluations before its potential use can be fully exploited. However, one important disadvantage of miR-155 as a diagnostic biomarker is that it is overexpressed not only in one specific malignancy but also in several, complicating diagnostic discriminations of the different malignancies. Noteworthily, an important advantage is the validated target genes of miR-155, which puts the biomarker into perspectives of molecular pathways.

Elevated miR-155 expression was generally associated with poor survival in both CLL and DLBCL, showing independent prognostic impact, though as a marker for the present prognostic tools (e.g., chromosomal subtyping and ABC/GCB) it did not add further information. In general, prognostic biomarkers only hold beneficial information, if nonresponsive patients can be treated differently. The biomarker then moves from prognostic to predictive, where it can be used to guide treatment choices. No thorough investigations have been reported of miR-155 as a predictive biomarker, though its prognostic observations could imply the need for new treatment options for patients with a high expression level. Akt inhibitors (currently in clinical trials [95]) have been suggested as efficient therapeutics for the treatment of patients with high miR-155 expression, since miR-155 activates this pathway. Logically, other novel treatments could evidently be anti-miRNAs suppressing the miR-155 expression and its oncogenic function. Their effect has been proved both* in vitro* and* in vivo*, and a targeted distribution model strengthens the potential as a novel therapeutic. At the present time, new clinical phase I trial of cutaneous T cell lymphoma (CTCL) investigates the safety and tolerability of anti-miR-155 (MRG-106) [96]. Presumably, this treatment might show interesting potential in DLBCL and CLL patients as well. Noteworthily, miravirsen, anti-miR-122, was the first microRNA targeted drug ever to reach clinical trials in 2009, for the management of hepatitis C viral infection [97]. Interestingly, miR-122 was later shown to be overexpressed in CTCL, suggesting that inhibition of miR-122 might also be a promising strategy in improving treatment outcome in these patients [98].

## 5. Conclusion

In summary, the expression of miR-155 shows potential as a diagnostic and prognostic biomarker, though further studies are warranted to assess its use in treatment prediction. Interestingly elevated expression was generally associated with poor treatment response, which is why it has been investigated and evidenced as an efficient therapeutic target. These properties prove that miR-155 has the potential to be a molecular tool in personalized medicine, bringing us one step closer to improvements of diagnosis and treatment.

## Supplementary Material

Table S1. Search strategy in PubMed and EMBASE PubMed and EMBASE were systemically searched for eligible articles using the listed search terms.
Figure S1. Flow diagram of study selection. The search for eligible articles from the databases PubMed and EMBASE was finalized November 18th 2015. The systematic search revealed 606 articles after removal of duplicates, which subsequently were manually screened based on title and abstract. Not English articles, reviews, and articles not focusing on B-cell malignancies, miR-155 as a biomarker or therapeutic target were excluded. Remaining articles were screened by full-text and 53 articles were included in the review.

## Figures and Tables

**Table 1:** Cancer relevant target genes for miR-155 supported by experimental observations.

Target genes	Main effect of aberrant miR-155 expression	Reference
SHIP1	↑ PI3K/AKT activity	[22, 23]
	↑ B-cell proliferation	
AID	↑ immunoglobulin diversification/class switch	[24]
PU.1	↑ immunoglobulin diversification/class switch	[25]
HGAL	↑ cell motility	[26]
C/EBP*β*	↑ B-cell proliferation	[23]
SMAD5	↑ evasion of TGF-*β*'s growth inhibitory effects	[27]
FADD	↓ apoptosis	[28]
Ripk	↓ apoptosis	[28]
SOCS1	↑ STAT5 activation	[29]

AID, activation-induced cytidine deaminase; C/EBP*β*, CCAAT/enhancer-binding protein *β*; FADD, Fas-Associated protein with Death Domain; HGAL, human germinal center-associated lymphoma; SHIP1, SH2 domain containing inositol 5′-phosphatase 1; SOCS1, suppressor of cytokine signaling protein 1.

**Table 2:** miR-155 as diagnostic biomarker in B-cell malignancies.

Disease	Sample type	Cohort		Initial miRNA selection	Method	Expression	Ref
DLBCL	FFPE	90 DLBCL cases 31 controls		Previous research	RT-qPCR	↑	[32]

DLBCL	CS	75 DLBCL cases 10 controls	T	Global screening	Microarray	↑	[46]
	FFPE	47 DLBCL cases 15 controls	V		RT-qPCR	↑	

DLBCL	Blood	20 DLBCL cases 20 controls	T	Previous research	RT-qPCR	↑	[37]
		75 DLBCL cases 77 controls	V			↑	

DLBCL	FFPE	80 DLBCL cases 12 controls		Global screening	Microarray	↑	[33]
		80 DLBCL cases 18 FL cases				NS	

DLBCL	FFPE	11 PCNSL cases 10 nDLBCL cases		Previous research	RT-qPCR	↑	[47]

DLBCL	CS, FFPE	35 DLBCL cases 12 controls		Global screening	RT-qPCR	↑	[34]
		35 DLBCL cases 27 FL cases				NS	

DLBCL	CS, FFPE	18 DLBCL cases 5 controls		Previous research	RNA-ISH	↑	[48]
					RT-qPCR	↑	

DLBCL	CS	29 DLBCL cases 12 BL cases	T	Global screening	Microarray	↑	[38]
	FFPE	43 DLBCL cases 28 BL cases	V		RT-qPCR	↑	

DLBCL	Blood	60 DLBCL cases 43 controls		Previous research	RT-qPCR	↑	[35]

DLBCL	FFPE	19 DLBCL cases 31 exN DLBCL cases		Previous research	RT-qPCR	NS	[49]

DLBCL	CS	22 DLBCL cases 7 controls		Previous research	RT-qPCR	↑	[50]

DLBCL	CS	79 DLBCL cases 36 BL cases		Global screening	Microarray	↑	[39]

DLBCL	CS	23 DLBCL cases 2 controls		Previous research	RT-qPCR	↑	[51]

DLBCL	FNAB	45 DLBCL cases 33 BL cases		Previous research	RT-qPCR	↑	[40]
		45 DLBCL cases 19 DLBCL/BL cases				↑	

DLBCL	FFPE	200 DLBCL cases 11 controls		Previous research	RT-qPCR	↑	[52]

DLBCL	CS	10 DLBCL EBV− cases 11 DLBCL EBV+ cases		Global screening	Sequencing	↑	[53]
					RT-qPCR	NS	

DLBCL	FFPE	58 DLBCL cases 7 controls		Global screening	RT-qPCR	↑	[36]
		58 DLBCL cases 46 FL cases				NS	

DLBCL	Ocular fluid	17 retinal DLBCL cases 12 uveitis cases		Global screening	RT-qPCR	↓	[54]

BL	CS	6 BL DLBCL (cell line)		Previous research	RT-qPCR	↓	[55]

BL	FFPE CS	3 BL cases 2 controls		Previous research	Northern blot	↓	[56]

BL	CS	11 BL cases 11 controls		Previous research	RT-qPCR	↓	[57]

BL	CS	12 BL cases 135 other L		Global screening	Microarray	↓	[38]

HL	FFPE	42 HL cases 8 controls		Global screening	Microarray	NS	[58]
					RT-qPCR	NS	

HL	CS	25 HL cases 7 controls		Previous research	RT-qPCR	↑	[50]

HL	CS, FFPE	5 HL cases 5 controls		Previous research	RT-qPCR	NS	[48]

MALT	CS	22 MALT cases 46 controls		Previous research	RT-qPCR	↑	[59]

MALT	CS	4 MALT cases 4 controls^*∗*^	T	Global screening	Microarray	↑	[60]
		14 MALT cases 14 controls^*∗*^	V		RT-qPCR	↑	

MALT	CS	3 MALT cases 3 controls^*∗*^	T	Global screening	Microarray	↑	[61]
		20 MALT cases 20 controls^*∗*^	V		RT-qPCR	↑	

FL	BM	5 FL cases 3 controls		Previous research	RT-qPCR	NS	[62]

SMZL	FFPE	15 SMZL cases 11 controls		Global screening	RT-qPCR	↑	[63]

SMZL	CS	31 SMZL cases 15 controls	T	Global screening	Microarray	↑	[64]
	FFPE	77 SMZL cases 6 controls	V		RT-qPCR	↑	

SMZL	CS FFPE	15 SMZL cases 9 controls		Global screening	Microarray	↑	[65]
					RT-qPCR	↑	

CLL	CS	70 CLL cases 18 controls		Previous research	RT-qPCR	↑	[66]

CLL	Blood	6 CLL cases 3 controls		Global screening	Microarray	↑	[67]

CLL	Blood	7 CLL cases 4 controls		Previous research	Northern blot	↑	[68]

CLL	Blood	69 CLL cases 15 controls		Global screening	Nanostring	↑	[69]
					RT-qPCR	↑	

CLL	Blood	113 CLL cases 7 controls		Previous research	RT-qPCR	↑	[70]

CLL	Blood	50 CLL cases 14 controls		Global screening	Microarray	↑	[71]
					RT-qPCR	↑	

CLL	Blood	38 CLL cases 9 controls		Global screening	Microarray	↑	[72]

CLL	Blood	56 CLL cases 7 controls		Previous research	RT-qPCR	↑	[73]

CLL	Blood	70 CLL cases 8 controls	T	Previous research	RT-qPCR	↑	[74]
		23 CLL cases 12 controls	V		RT-qPCR	↑	

*Disease*: DLBCL, diffuse large B-cell lymphoma; BL, Burkitt's lymphoma; HL, Hodgkin lymphoma; MALT, mucosa-associated lymphoid tissue; FL, follicular lymphoma; SMZL, splenic marginal zone lymphoma; CLL, chronic lymphocytic leukemia. *Sample type*: FFPE, formalin-fixed paraffin-embedded tissue samples; CS, clinical samples; FNABs, fine needle aspirations. *Cohort*: controls, nonmalignant tissues; PCNSL, primary CNS lymphoma; nDLBCL, nodal DLBCL; exN DLBCL, extranodal DLBCL; EBV, Epstein-Barr virus; L, lymphoma; control^*∗*^, adjacent normal tissue, T; training set; V, validation set. *Method*: RT-qPCR, reverse transcription quantitative PCR; RNA-ISH, RNA *in situ* hybridization. *Expression*: ↑, increased; ↓, decreased; NS, not significant. *Ref*: reference.

**Table 3:** miR-155 as prognostic biomarker in diffuse large B-cell lymphoma (DLBCL), its expression in relation to GCB or nGCB/ABC subtyping, and direct relation to prognosis.

Cohort	Molecular subtype	Sample type	Initial miR selection	Method	Outcome			Ref
					Prognosis	Measure	Molecular subtype	
90 DLBCL (51/39)	21 GCB 69 nGCB	FFPE	Previous research	RT-qPCR	↑ poor	RA, OR	↑ nGCB	[32]

	20 GCB 34 nGCB	FFPE	Global screening	RT-qPCR			↑ nGCB	[46]

	32 GCB 28 nGCB	FFPE	Global screening	Microarray			↑ nGCB	[33]

	9 GCB 12 nGCB	FFPE	Previous research	RT-qPCR			NS	[47]

	85 GCB 34 ABC	CS	Previous research	Microarray			↑ ABC	[89]

	17 GCB 18 ABC	CS, FFPE	Global screening	RT-qPCR			↑ ABC	[34]

	11 GCB 7 ABC	CS, FFPE	Previous research	ISH RT-qPCR			↑ ABC	[48]

	9 GCB 9 nGCB	FFPE	Previous research	ISH Microarray			NS	[90]

	8 GCB 15 nGCB	CS	Previous research	RT-qPCR			NS	[50]

54 DLBCL (27/27)	32 GCB 27 ABC	CS	Global screening	Microarray	↑ poor	EFS	↑ ABC	[39]

	4 GCB 19 ABC	CS	Previous research	RT-qPCR			↑ ABC	[51]

129 DLBCL		CS	Previous research		NS	PFS, OS		[75]
24 ABC					↑ improved	PFS, OS		

53 DLBCL	25 GCB 25 nGCB	FFPE	Previous research	RT-qPCR	NS	EFS, OS	↑ nGCB	[36]

	14 GCB 36 nGCB	FFPE	Previous research	RT-qPCR			NS	[49]

200 DLBCL (121/79)		FFPE	Previous research		NS	PFS, OS		[52]

( / ) in column 1: number of patients with high and low miR-155 expression; *numbers* in column 2 indicate how many DLBCL patients included for miR-155 expression evaluation in each molecular subtype; *subtype*: GCB, germinal center B-cell-like; nGCB, non-GCB; ABC, activated B-cell-like. *Sample type*: FFPE, formalin-fixed paraffin-embedded tissue samples; CS, clinical samples. *Method*: RT-qPCR, reverse transcription quantitative PCR; ISH, *in situ* hybridization. *Prognosis*: ↑, increased expression; ↓, decreased expression; NS, not significant. *Outcome measure*: RA, response assessment; OR, overall response; EFS, event-free survival; PFS, progression-free survival; OS, overall survival. *Ref*: reference.

**Table 4:** miR-155 as prognostic biomarker in CLL, its expression in relation to established prognostic factors, and direct relation to prognosis.

Cohort CLL		Sample type	Initial miR selection	Method	Outcome^*∗*^				Ref
					Unfavorable factors	Favorable factors	Prognosis	Measure	
70		CS	Previous research	RT-qPCR	↑	↑			[66]

109 (56/53)		CS	Previous research	nCounter	↑	↓	↑ poor	OS PFS	[79]
9 (4/5)		Blood					↑ poor	Relapse	

8 (4/4)		Blood	Previous research	Northern blot		NS			[68]

43 (18/25)		Blood	Previous research	RT-qPCR	NS	NS			[70]

70	T	Blood	Global screening	Microarray		↑			[91]
24	V			RT-qPCR		↑			

70 (33/37)		FFPE Blood	Previous research	RT-qPCR	NS	NS			[80]

50		Blood	Global screening	Microarray	NS	NS			[71]

143		Blood	Previous research	RT-qPCR	NS	NS	↑ poor	RA OS	[74]
85 (31/45)							↑ poor	RA	

86 (55/31)	T	Blood	Previous research	RT-qPCR	↑	↓	↑ poor	TFS	[81]
181 (95/86)	V			Microarray	↑	↓	↑ poor	TFS	

94	T	CS	Global screening	Microarray	↑		↑ poor	TIT	[92]
50	V				↑				

61	T	CS	Global screening	Microarray RT-qPCR	↑	↓			[77]
29	V				↑	↓			

104 (64/40)		Blood	Previous research	RT-qPCR	↑		NS	OS PFS	[78]

56		Blood	Previous research	RT-qPCR	NS	NS			[73]

28		Blood	Global screening	Microarray	NS	NS	NS	TIT	[72]

*Cohort*: T, training set; V, validation set. *Sample type*: CS, clinical sample; FFPE, formalin-fixed paraffin-embedded tissue samples. *Method*: RT-qPCR, reverse transcription quantitative PCR. *Outcome*: ^*∗*^unfavorable factors: 17p deletion, 11q deletion, trisomy 12, CD38^+^ cells, ZAP-70 expression > 20%, and/or advanced disease stage determined by either Rai or Binet. Favorable factors: normal karyotype, IgHV mutations, and/or 13q deletion [76]; ↑, increased expression; ↓, decreased expression; NS, not significant. *Outcome measure*: OS, overall survival; PFS, progression-free survival; RA, response assessment; TFS, treatment-free survival; TI, time to initial treatment. *Ref*: reference.
