# Supplementary material for: miR-155 as a Biomarker in B-Cell Malignancies
Source: Biomed Res Int. 2016 May 16;2016:9513037. doi: 10.1155/2016/9513037 (PMC4884835; doi:10.1155/2016/9513037)

Supplementary

Table S1. Search strategy in PubMed and EMBASE

Database	PubMed	EMBASE
**Search Terms**	#1: "Lymphoma"[Mesh]<br>#2: lymphoma*<br>#3: "b-lymphocytes"[MeSH Terms] OR "b-lymphocytes" OR "B-cell"<br>#4: "Lymphoma, B-Cell"[Mesh]<br>#5: burkitt[tw]<br>#6: #1 OR #2 OR #3 OR #4 OR #5<br>#7: rno-miR-155 OR rno miR 155 OR rno-miR 155 5p OR rno-miR-155-5p<br>#8: mmu-mir-155 OR mmu mir 155 OR Mir155<br>#9: miR 155 OR miRNA 155 OR hsa mir 155 OR microRNA 155<br>#10: miR-155 OR miRNA 155 OR hsa-mir-155 OR microRNA-155<br>#11: "MIRN155 microRNA, human"[Supplementary Concept] OR "MIRN155 microRNA, rat"[Supplementary Concept] OR "Mirn155 microRNA, mouse"[Supplementary Concept]<br>#12: #7 OR #8 OR #9 OR #10 OR #11<br>#13: #6 OR #12<br>No filters	#1: microRNA 155/<br>#2: (rno-miR-155 or rno miR 155 or rno-miR 155 5p or rno-miR-155-5p or mmu-mir-155 or mmu mir 155 or Mir155 or miR 155 or miRNA 155 or hsa mir 155 or microRNA 155 or miR-155 or miRNA 155 or hsa-mir-155 or microRNA-155).mp.<br>#3: #1 or #2<br>#4: exp B lymphocyte/<br>#5: B-cell lymphoma/<br>#6: (b-lymphocytes or B-cell).mp.<br>#7: exp lymphoma/<br>#8: lymphoma*.mp.<br>#9: or/#4-8<br>#10: #3 and #9<br>No filters

Figure S1. Flow diagram of study selection

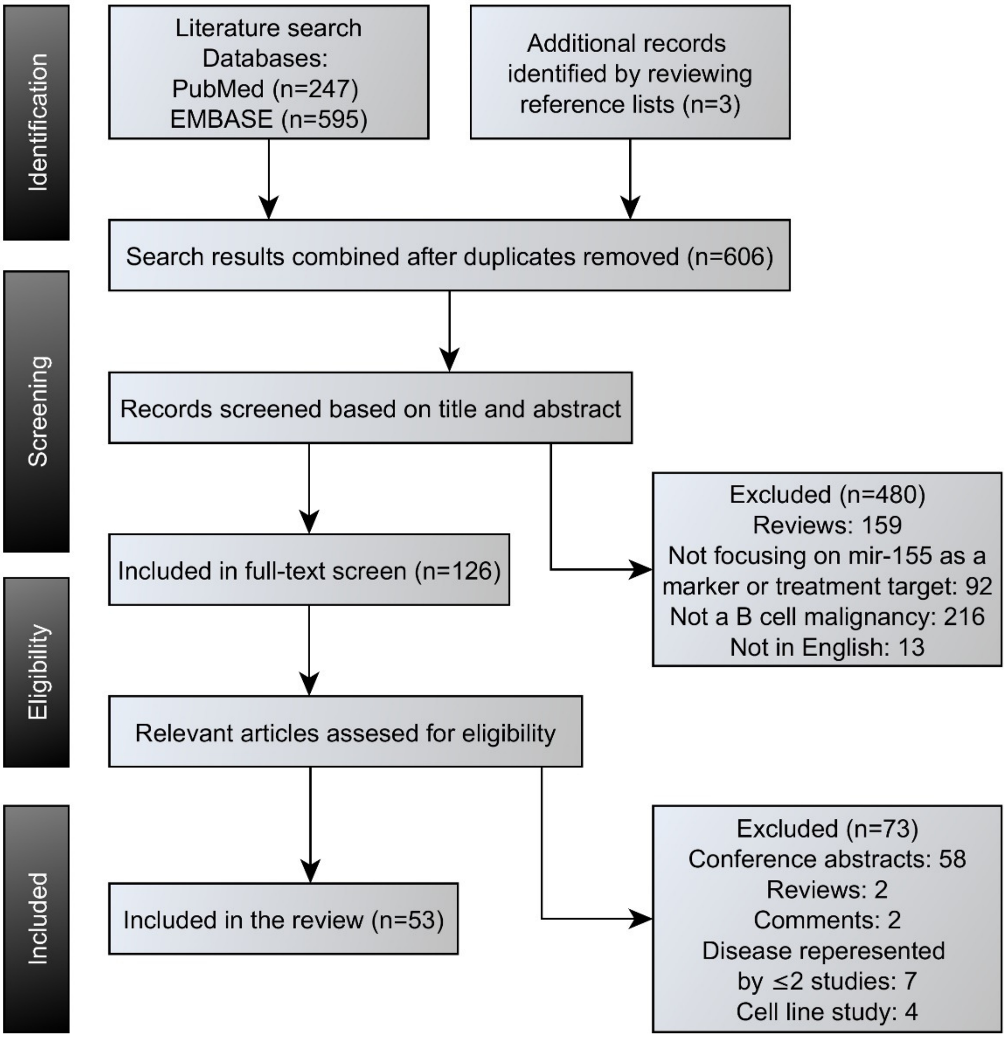

Supplement: Supplementary file 1 — Table S1. Search strategy in PubMed and EMBASE PubMed and EMBASE were systemically searched for eligible articles using the listed search terms. Figure S1. Flow diagram of study selection. The search for eligible articles from the databases PubMed and EMBASE was finalized November 18th 2015. The systematic search revealed 606 articles after removal of duplicates, which subsequently were manually screened based on title and abstract. Not English articles, reviews, and articles not focusing on B-cell malignancies, miR-155 as a biomarker or therapeutic target were excluded. Remaining articles were screened by full-text and 53 articles were included in the review. [file 9513037.f1.pdf]
